# The relationship between proactive health literacy and lifestyle behaviors among residents in a region of China

**DOI:** 10.3389/fpubh.2026.1777008

**Published:** 2026-02-19

**Authors:** Tingting Wang, Rong Huang, Feng Zhang, Enhong Dong, Haonan Shi, Wandi Li, Meina Yan, Xuan Yao, Yanmei Wang

**Affiliations:** 1 Department of Nursing, Pudong Gongli Hospital, Shanghai University of Medicine & Health Sciences, Shanghai, China; 2 School of Nursing & Health Management, Shanghai University of Medicine & Health Sciences, Shanghai, China; 3 School of Humanities, Zhuhai City Polytechnic, Zhuhai, China; 4 School of Medicine, Shanghai University, Shanghai, China; 5 Kangqiao Health Service Center, Shanghai, China

**Keywords:** China, health literacy, health promotion, healthy lifestyle, proactive health

## Abstract

**Objectives:**

With the rapid pace of modern lifestyles, unhealthy behaviors such as smoking, excessive drinking, poor diet, and physical inactivity lead to increased health risks and a greater burden of chronic diseases. Proactive health, which emphasizes individual initiative in health management, is crucial for disease prevention. This study explores the relationship between proactive health literacy (PHL) and lifestyle behaviors among community residents.

**Methods:**

A cross-sectional survey was conducted among 560 community residents in Anhui Province, China, using a self-designed questionnaire. Data regarding PHL (knowledge, beliefs, and behaviors), lifestyle behaviors (smoking, drinking, diet, and exercise), and self-rated health were collected. Descriptive statistics, correlation analysis (Spearman’s rho), and multiple linear regression analysis were performed.

**Results:**

Among the dimensions of health literacy, knowledge scores were relatively low (median = 3.62), belief scores were high (median = 4.00), and behavior scores were in the intermediate range (median = 3.91). Correlation analysis showed that health literacy was negatively correlated with age (*r* = −0.169, *p* < 0.05), smoking (*r* = −0.150, *p* < 0.05), and drinking (*r* = −0.103, *p* < 0.05) and positively correlated with education (*r* = 0.192, *p* < 0.05) and monthly income (*r* = 0.134, *p* < 0.05). Multiple linear regression analysis indicated that female sex (*β* = 0.242, *p* < 0.05), higher monthly income (*β* = 0.123, *p* < 0.05), better self-rated health (*β* = −0.181, *p* < 0.05), and healthier dietary habits (*β* = −0.134, *p* < 0.05) were significant predictors of higher overall PHL.

**Conclusion:**

Residents’ health knowledge was insufficient, requiring strengthened health education, particularly for older adults and those with lower levels of education/income. Although health beliefs are strong, transforming them into sustained healthy actions remains a challenge. Health literacy is closely linked to lifestyle behaviors, underscoring the need for targeted interventions to promote proactive health management.

## 1 Introduction

Unhealthy lifestyle behaviors—such as smoking, alcohol consumption, poor diet, and insufficient physical activity—are major, modifiable contributors to the burden of non-communicable diseases (NCDs) and related disabilities. Addressing these changeable risk factors is widely regarded as a key strategy for reducing premature mortality and health loss (1). At the same time, lifestyle behaviors commonly exhibit “clustering/co-occurrence”: Multiple behaviors (e.g., physical activity/sedentary time, sleep, diet quality, smoking, and drinking) often combine into distinct behavioral patterns at the individual level, generating cumulative risk and increasing the complexity of community-based interventions (2). In China, large-scale studies among young people have similarly identified clear clustering of healthy lifestyle behaviors and their associations with outcomes such as mental health, underscoring the need for a “multi-behavior” perspective to support more targeted health promotion strategies (3).

Health literacy is widely viewed as an upstream determinant of health behaviors and outcomes. Recent evidence synthesis suggests an overall positive association between health literacy and health-promoting lifestyle behaviors, although the strength of this association may be modest and shaped by sociodemographic factors (4). A cross-sectional study in Japan by Kinoshita et al. further indicated that different levels of health literacy (functional, communicative/interactive, and critical) are associated with health-related lifestyle behaviors, providing empirical support for a “literacy–behavior” pathway (5). Similarly, a survey in China by Ma et al. (6) found that health literacy was related to a health-promoting lifestyle and might operate through pathways such as family health and physical activity, offering practical implications for community-based health education and behavioral interventions. In addition, Zhang et al. (7) examined the association between different types of health literacy and health-promoting lifestyles, highlighting the importance of stratified health education and the need for tailored interventions.

Despite the accumulating evidence supporting links between health literacy and lifestyle behaviors, the majority of existing studies focus on traditional health literacy domains and often overlook the action-oriented capabilities associated with a “proactive health” approach. This includes residents’ capacity to actively seek information, form health beliefs, and consistently translate them into daily practices (8). Moreover, since lifestyle behaviors tend to cluster, studies that focus on single behaviors may underestimate real-world intervention needs, and some research has not fully incorporated multi-behavior combinations or behavioral-pattern frameworks (2, 3). Evidence synthesis also suggests that the overall association between health literacy and health-promoting lifestyle behaviors may be relatively weak, implying that improving knowledge-based “cognitive literacy” alone may be insufficient to drive behavior change; greater attention is needed on action orientation, practicality, and contextual relevance (4). Accordingly, in Chinese community settings, there remains a clear need to evaluate how health literacy relates to multiple lifestyle behaviors using a construct closer to “proactive health management” to generate more actionable evidence for stratified interventions.

Against this background, we conducted a cross-sectional survey among community residents in a region of China to examine “proactive health literacy (PHL)” and core lifestyle behaviors, including smoking, drinking, diet, and physical activity. We aimed to describe residents’ PHL and its component dimensions, characterize key lifestyle behaviors, and assess the associations between PHL and lifestyle behaviors, including potential heterogeneity across population subgroups. A distinctive feature of this study is its proactive approach to health: It extends beyond the traditional emphasis on understanding health information to a more action-oriented capacity—“proactive acquisition, belief formation, and translation into practice”—which aligns closely with residents’ self-management needs in the context of China’s health strategy (8). In addition, by explicitly adopting a multi-behavior perspective, the study aimed to identify more tractable leverage points within behavioral clustering and real-world complexity, thereby informing stratified health education, intervention design, and resource allocation at the community level.

## 2 Methods

### 2.1 Data sources and preprocessing

#### 2.1.1 Participants

Residents were recruited from a community in Anhui Province, China.

The inclusion criteria were as follows: (1) Must be Permanent residents of the community (local residence ≥6 months), (2) must provide informed consent, and (3) must have normal mental status along with sufficient communication and reading abilities.

The exclusion criteria were as follows: (1) Cognitive impairment and (2) severe visual, hearing, or reading impairments.

#### 2.1.2 Sampling and sample size

**Figure 1:**
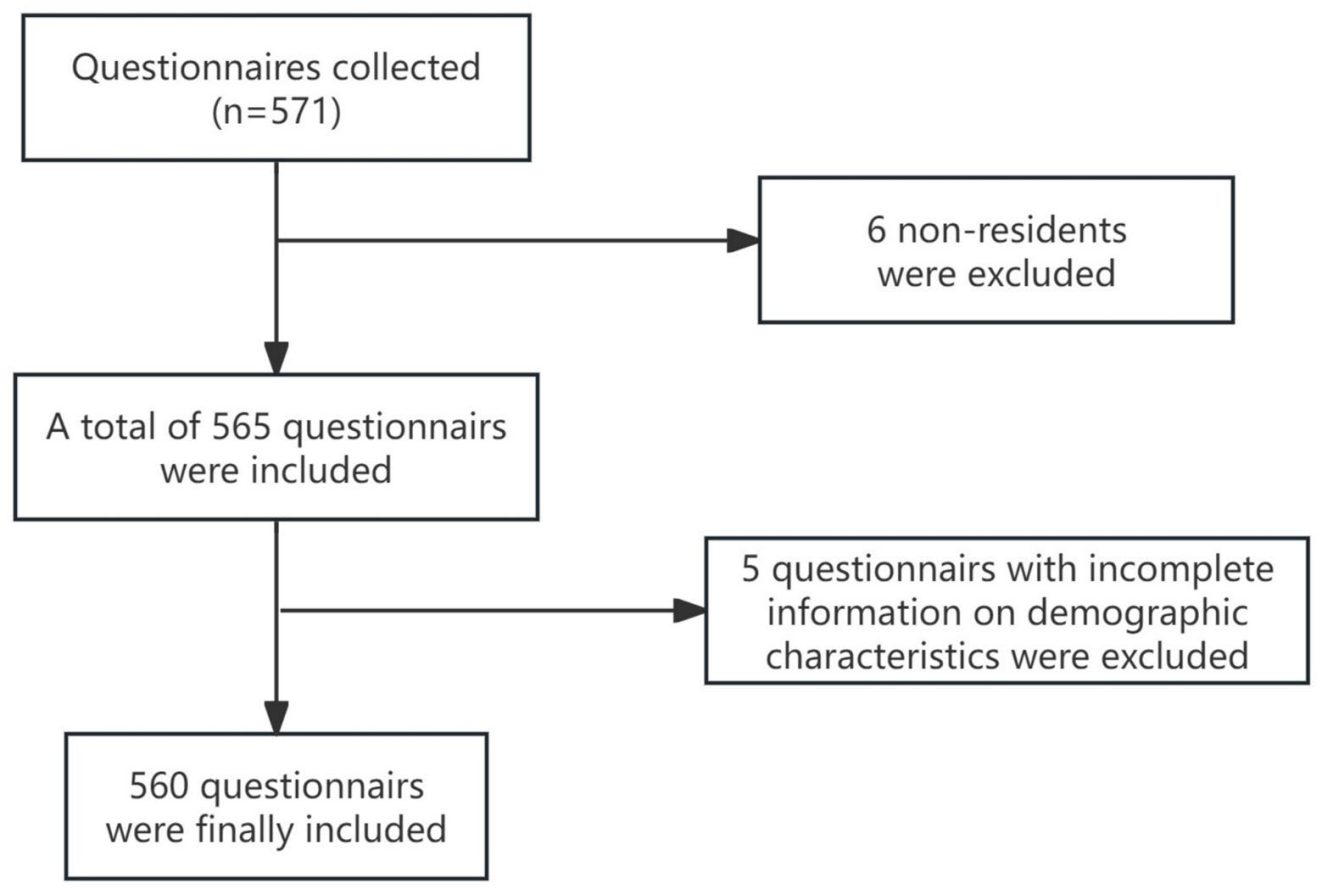
Flowchart of this study.

We conducted a cross-sectional survey using simple random sampling, employing both on-site and online questionnaires. The sample size was calculated using the formula: *n* = *Z*^2^*p*(1 − *p*)/*d*^2^, assuming a 75.7% national prevalence of unhealthy habits, *Z* = 1.96, and *d* = 0.05, resulting in a minimum sample size of 283. To account for 10% of non-eligible/invalid responses, the target sample size was set to be greater than 311. We collected 571 questionnaires. However, two respondents refused to provide informed consent, four were non-permanent residents, and five had incomplete key information, leaving 560 participants for analysis (effective response rate 98.07%) (see Figure 1).

### 2.2 Measures and data collection

A total of two instruments were used in this cross-sectional survey: ① The Lifestyle Behaviors Questionnaire and ② the PHL and Competency Assessment Questionnaire. Both instruments were developed by the authors for the present study in 2024 to suit community-based administration in China.

Lifestyle behaviors were assessed using the Lifestyle Behaviors Questionnaire, which captured major domains relevant to NCD prevention, including smoking, alcohol consumption, dietary habits, and physical activity. As these items represent distinct behaviors rather than a single latent construct, internal consistency indices (e.g., Cronbach’s *α*) were not applicable; the items were analyzed as separate behavioral indicators.

The PHL was assessed using the PHL and Competency Assessment Questionnaire, developed by the authors in 2024. The questionnaire comprises 36 items covering three dimensions—health knowledge, health beliefs, and health behaviors—rated on a 5-point Likert scale [1–5]. After reverse-scoring negatively worded items, dimension scores and a total score were calculated, with higher scores indicating higher PHL (total score range: 36–180).

Reliability and validity of the PHL questionnaire were evaluated in the study sample. Internal consistency was high (Cronbach’s *α* = 0.948; standardized *α* = 0.949; 36 items; Table 1), which was expected given that the questionnaire was iteratively refined through multiple rounds of expert Delphi consultation and pilot testing to improve item clarity and coherence. We also confirmed that there were no verbatim repeated items; nevertheless, an alpha value this high may partly reflect overlap among conceptually similar items, and future validation could consider item reduction or short-form development while preserving content coverage. The Kaiser–Meyer–Olkin measure of sampling adequacy was 0.944, and Bartlett’s test of sphericity was significant (*χ*^2^ = 14758.717; df = 666; *p* < 0.0001; Table 2), supporting the adequacy of the item correlation matrix for factor analytic assessment of construct validity.

**Table 1:** Cronbach’s alpha reliability statistics.

Cronbach’s alpha	Cronbach’s alpha (based on standardized items)	Number of items
0.948	0.949	36

**Table 2:** Kaiser–Meyer–Olkin measure and Bartlett’s test of sphericity.

Measure/test	Statistic	Value
Kaiser–Meyer–Olkin (KMO) measure of sampling adequacy	—	0.944
Bartlett’s test of sphericity	Approximate *χ*^2^	14758.717
	df	666
	*P*-value	<0.0001

### 2.3 Data analysis

Data were entered in Excel and analyzed using SPSS 20.0. Descriptive statistics (frequencies, percentages, medians, and interquartile ranges) were used to summarize the sample and key variables. Spearman’s rank correlation coefficient was used to analyze relationships between variables. Multiple linear regression analyses were conducted to identify factors associated with health literacy dimension scores and the total score. Statistical significance was set at a *p*-value of <0.05.

Variable coding: For Spearman correlations and multiple linear regression analyses, categorical variables were recoded as ordinal numeric variables to facilitate the interpretation of coefficients. Higher values represented older age, higher education and income, poorer self-rated health, greater tobacco and alcohol consumption, a more meat-based dietary pattern, and more regular meals. Coding was as follows: Sex (0 = male and 1 = female), age group (1 = 18–35, 2 = 36–59, and 3 = ≥60), education level (1 = junior secondary school or below, 2 = senior secondary/secondary vocational school, 3 = college/associate degree, 4 = university/bachelor’s degree, and 5 = postgraduate or above), monthly household income (1 = <500 CNY, 2 = 500–999 CNY, 3 = 1,000–1999 CNY, 4 = 2000–4,999 CNY, 5 = 5,000–9,999 CNY, and 6 = ≥10,000 CNY), self-rated health (1 = very good to 5 = very poor), smoking status (0 = non-smoker without passive exposure, 1 = non-smoker with passive exposure, 2 = former smoker, and 3 = current smoker), alcohol consumption in the past year (0 = non-drinker, 1 = former drinker, 2 = <1/month, 3 = 1–10/month, and 4= > 10/month), dietary habits (dietary pattern: 0 = balanced/mixed diet, 1 = predominantly plant-based, and 2 = predominantly meat-based), and meal regularity (0 = irregular, 1 = usually skip breakfast, and 2 = regular meals).

### 2.4 Quality control

Strict inclusion/exclusion criteria were applied. Investigators explained the study, ensured confidentiality, and obtained consent. For on-site surveys, investigators were available for clarification. Data entry was double-checked for accuracy.

The studies involving human participants were reviewed and approved by the Ethics Committee of Shanghai University of Medicine & Health Sciences (SUMHS) (Approval No.: 2024-HG-WTT). All participants provided written informed consent prior to participation.

## 3 Results

### 3.1 Sample characteristics

Among the 560 participants, 54.5% were male and 45.5% were female. The largest age group was ≥60 years (40.4%), followed by 36–59 years (38.4%). The majority of participants had an education level of junior high school or below (63.0%). Monthly personal/household income was mostly 2000–5,000 RMB (32.1%). Regarding self-rated health, 25.0% of participants reported “very good,” 41.1% “good,” 27.1% “fair,” 5.9% “poor,” and 0.9% “very poor.” Sociodemographic characteristics of participants are presented in Table 3.

**Table 3:** Sociodemographic characteristics of participants.

Characteristic	Category	*n*	%
Sex	Male	305	54.5
	Female	255	45.5
Age group (years)	<35	119	21.3
	36–59	215	38.4
	≥60	226	40.4
Education level	Junior secondary school or below	353	63.0
	Senior secondary school or secondary vocational school	83	14.8
	College (associate degree)	27	4.8
	University (bachelor’s degree)	86	15.4
	Postgraduate or above	11	2.0
Monthly household income (participant and spouse only)*	<500 CNY	74	13.2
	500–999 CNY	46	8.2
	1,000–1999 CNY	122	21.8
	2000–4,999 CNY	180	32.1
	5,000–9,999 CNY	101	18.0
	≥10,000 CNY	37	6.6
Self-rated health in the past year	Very good	140	25.0
	Good	230	41.1
	Fair	152	27.1
	Poor	33	5.9
	Very poor	5	0.9
Total	—	560	100.0

*Income includes only the participant and spouse (if applicable). CNY = Chinese yuan.

### 3.2 Lifestyle behaviors

**Figure 2:**
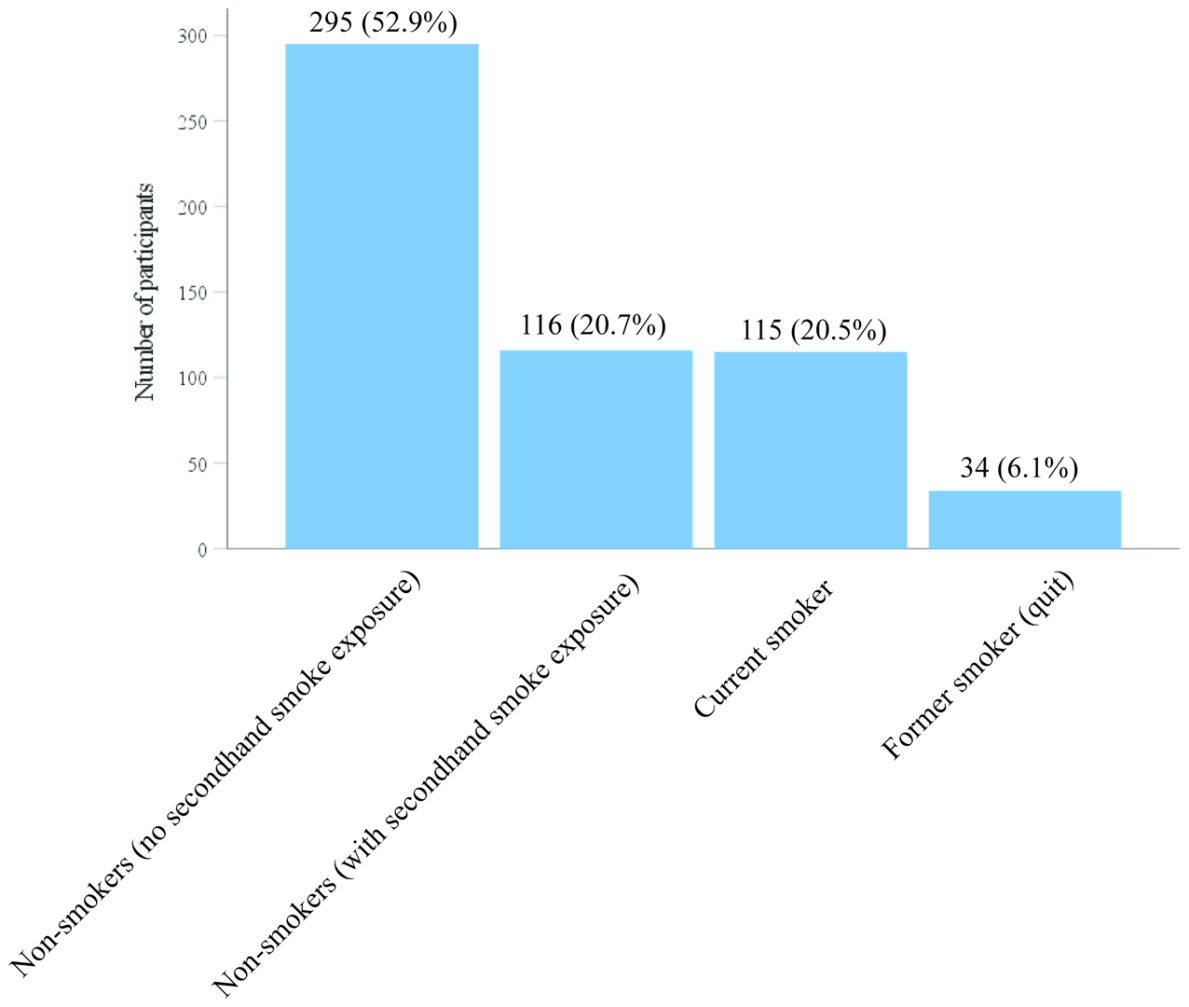
Smoking status of participants.

Smoking: The current smoking rate was 20.5% (*n* = 115), while the smoking cessation rate was 6.1% (*n* = 34). Among the non-smokers, 20.7% (*n* = 116) reported exposure to secondhand smoke (Figure 2).

**Figure 3:**
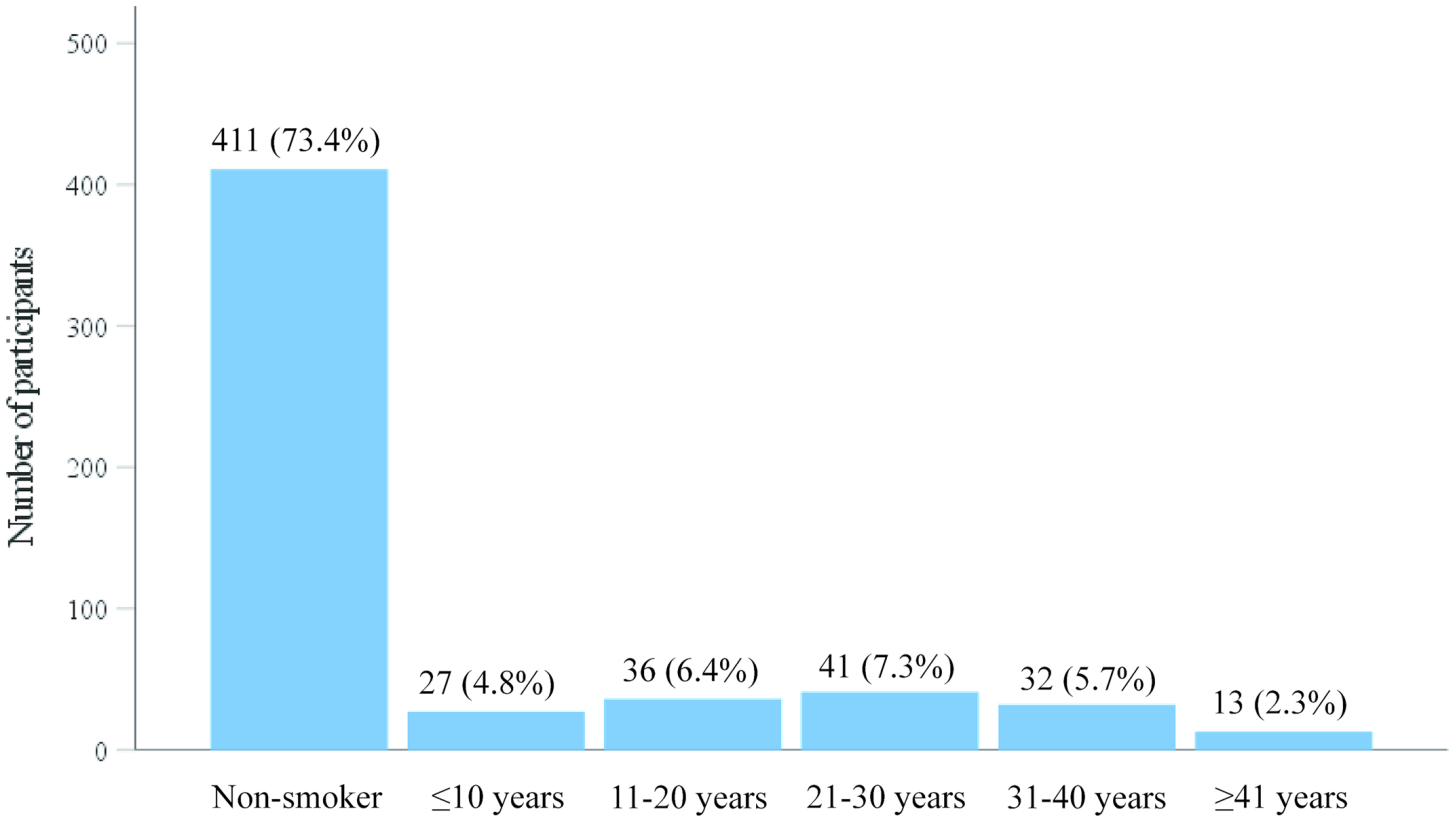
Smoking duration among participants.

In addition, there were 13 people who had smoked for 41 years or more, accounting for 2.3% of participants; 32 people who had smoked for 31–40 years, accounting for 5.7%; 41 people who had smoked for 21–30 years, accounting for 7.3%; 36 people who had smoked for 11–20 years, accounting for 6.4%; and 27 people who had smoked for 10 years or less, accounting for 4.9%. Long-term smokers (≥21 years) accounted for 15.3% of participants (7.3% + 5.7% + 2.3%) and were considered to have a relatively high health risk. The duration of smoking among the residents showed a right-skewed distribution, with more smokers in the young and middle-aged groups and relatively fewer individuals who had smoked for more than 41 years (Figure 3).

**Figure 4:**
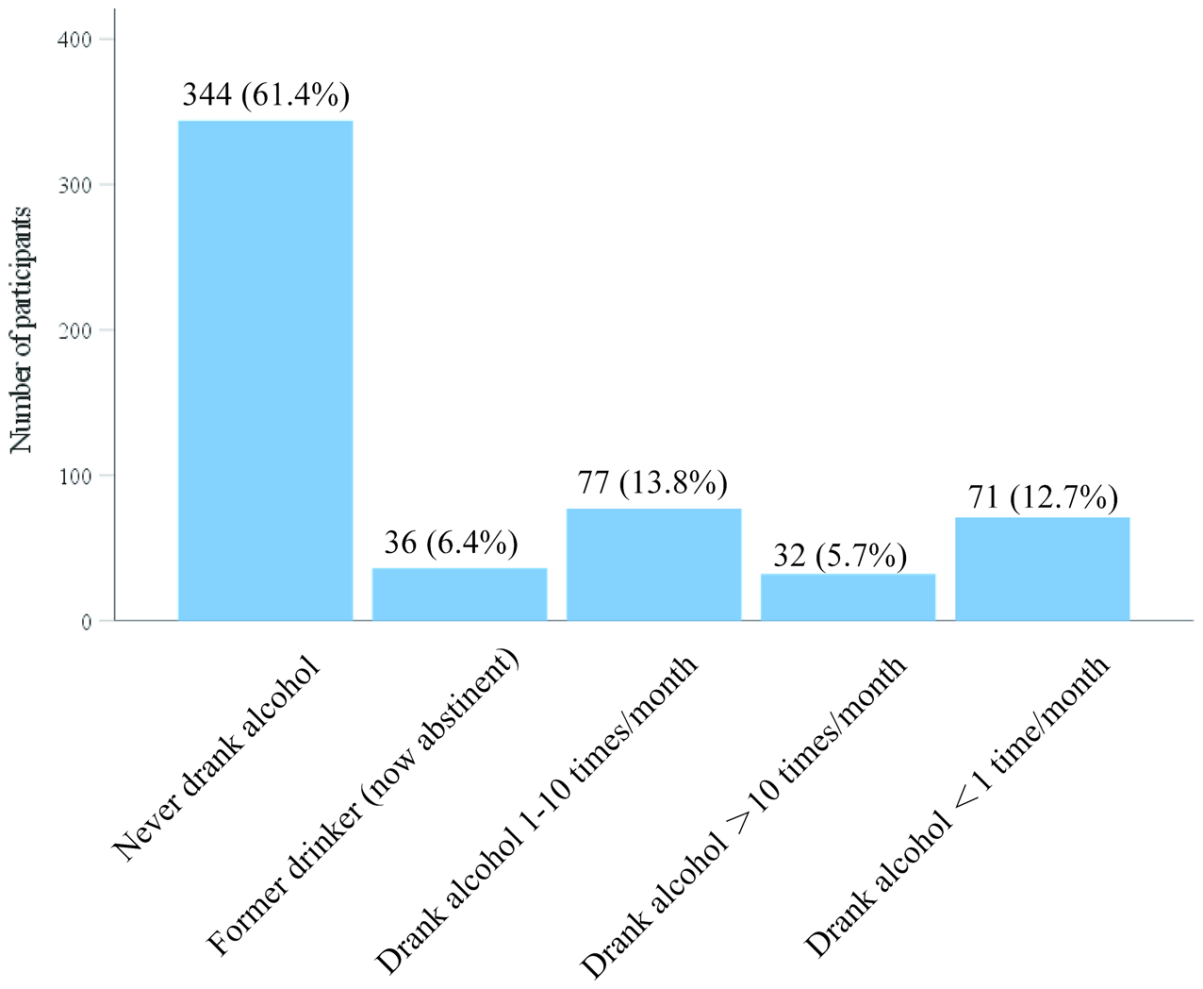
Alcohol consumption status of participants.

Drinking: Among residents, 61.4% had never consumed alcohol, representing the largest group. A total of 6.4% reported having drunk alcohol before but had since quit. The current drinking rate was 32.2%, with 12.7, 13.8, and 5.7% of residents consuming alcohol at different frequencies, including 5.7% who drank more than 10 times per month (Figure 4).

The current drinking rate was 32.2%. The abstinence rate was 6.4%. Among drinkers, spirits were the most commonly consumed type of alcohol (55.1%), followed by beer (35.2%). The types of alcoholic beverages consumed are presented in Table 4.

**Table 4:** Types of alcoholic beverages consumed among drinkers.

Beverage type	*n*	%
Spirits (distilled liquor)	119	55.1
Beer	76	35.2
Wine, rice wine, or yellow wine	14	6.5
Other	7	3.2
Total	216	100.0

Analysis of the data in Table 5 showed that among drinkers, spirits and beer were the primary types of alcohol consumed. Beer was more popular among individuals with moderate to low drinking frequency, while spirits were preferred by those with higher drinking frequency. The number of participants who consumed wine, rice wine, and yellow wine was relatively small, and these beverages were mainly consumed by individuals with lower drinking frequency. Former drinkers primarily consumed spirits, with 72.2% of them having a history of drinking spirits.

**Table 5:** Cross-tabulation of drinking frequency and main type of alcoholic beverage consumed.

Drinking status in the past year	Non-drinker	Spirits (distilled liquor)	Beer	Wine/rice wine/yellow wine	Other	Total
Never drank alcohol	344	0	0	0	0	344
Former drinker (drank previously, now abstinent)	0	26	8	2	0	36
Drank alcohol, less than once per month	0	21	42	5	3	71
Drank alcohol, 1–10 times per month	0	45	24	6	2	77
Drank alcohol, >10 times per month	0	27	2	1	2	32
Total	344	119	76	14	7	560

Data are presented as *n*. “Main type of alcoholic beverage” refers to the beverage most commonly consumed by respondents who reported drinking.

Diet: Among participants, 77.0% reported following a balanced meat–vegetable diet, 17.5% adhered to a vegetarian-based diet, and 5.5% adhered to a meat-based diet. A total of 40.5% reported a preference for high-salt, high-sugar, high-fat, or spicy foods. In addition, 88.6% reported regular meal times. Dietary patterns of participants are presented in Table 6.

**Table 6:** Dietary patterns of participants.

Domain	Category	*n*	%
Dietary pattern	Predominantly meat-based	31	5.5
	Balanced (mixed diet)	431	77.0
	Predominantly plant-based	98	17.5
Taste preference	Light (less oily/salty/sweet)	333	59.5
	Other (e.g., high sugar, oily, salty, or spicy)	227	40.5
Meal regularity	Usually skip breakfast	37	6.6
	Regular meals (three meals on time)	496	88.6
	Irregular	27	4.8
Total	—	560	100.0

Physical activity: Among participants, 42.5% reported no vigorous physical activity in the past week. Only 24.6% met the WHO recommendation of ≥3 sessions of vigorous activity per week. In addition, 70% reported moderate activity, while 30% reported no activity. Walking was highly prevalent, with 93.8% walking for at least 10 min at a time. Regarding sedentary behavior, 73% reported daily sedentary time of ≥4 h, and 7.8% exceeded 8 h per day. Physical activity patterns of participants are presented in Table 7.

**Table 7:** Physical activity patterns of participants.

Item	Response category	*n*	%
In the past 7 days, on how many days did you engage in vigorous physical activity (e.g., carrying heavy loads, digging, aerobic exercise, or fast cycling)?	>5 times/week	70	12.5
	1–2 times/week	184	32.9
	3–5 times/week	68	12.1
	No vigorous physical activity	238	42.5
In the past 7 days, on how many days did you engage in moderate physical activity (e.g., carrying light loads, cycling at a regular pace, or playing badminton or table tennis; walking not included)?	>5 times/week	70	12.5
	1–2 times/week	206	36.8
	3–5 times/week	116	20.7
	No moderate physical activity	168	30.0
In the past 7 days, on how many days did you walk for at least 10 min at a time?	>5 times/week	197	35.2
	1–2 times/week	167	29.8
	3–5 times/week	161	28.8
	No walking	35	6.3
On workdays in the past 7 days, how many hours per day did you spend sitting on average?	<4 h/day	151	27.0
	4–8 h/day	365	65.2
	>8 h/day	44	7.8
Total	—	560	100.0

### 3.3 Proactive health literacy

Median scores were as follows: Knowledge = 3.62 (IQR: 3.15–4.00), beliefs = 4.00 (IQR: 3.58–4.08), behaviors = 3.91 (IQR: 3.45–4.00), and total = 4.15 (IQR: 3.74–4.36). Knowledge scores were relatively lower than belief and behavior scores. The scores of PHL are presented in Table 8.

**Table 8:** Scores of proactive health literacy.

Dimension	Q1	Median	Q3
Knowledge (mean score)	3.15	3.62	4.00
Beliefs (mean score)	3.58	4.00	4.08
Behaviors (mean score)	3.45	3.91	4.00
Overall proactive health literacy (mean score)	3.74	4.15	4.36

### 3.4 Correlation analysis (Spearman’s rho)

Health literacy (total score) was significantly negatively correlated with age (*r* = −0.169, *p* < 0.05), smoking (*r* = −0.150, *p* < 0.05), drinking (*r* = −0.103, *p* < 0.05), and unhealthy dietary habits (*r* = −0.140, *p* < 0.05). It was positively correlated with education level (*r* = 0.192, *p* < 0.05) and monthly income (*r* = 0.134, *p* < 0.05). Self-rated health showed a negative correlation with health literacy (*r* = −0.272, *p* < 0.05). Smoking and drinking were positively correlated (*r* = 0.325, *p* < 0.05). Sex was negatively correlated with smoking (*r* = −0.465, *p* < 0.05) and drinking (*r* = −0.426, *p* < 0.05). Spearman’s rank correlations between sociodemographic characteristics, lifestyle factors, and PHL are presented in Table 9.

**Table 9:** Spearman’s rank correlations between sociodemographic characteristics, lifestyle factors, and proactive health literacy.

Variable	Sex	Age group	Education level	Monthly household income	Self-rated health (past year)	Smoking status	Alcohol consumption (past year)	Dietary habits	Meal regularity	Proactive health literacy
Sex	1.000	—	—	—	—	—	—	—	—	—
Age group	−0.115**	1.000	—	—	—	—	—	—	—	—
Education level	0.013	−0.567**	1.000	—	—	—	—	—	—	—
Monthly household income	−0.172**	−0.247**	0.171**	1.000	—	—	—	—	—	—
Self-rated health (past year)	0.052	0.346**	−0.307**	−0.234**	1.000	—	—	—	—	—
Smoking status	−0.465**	0.174**	−0.115**	0.024	0.060	1.000	—	—	—	—
Alcohol consumption (past year)	−0.426**	−0.055	0.082	0.081	0.076	0.325**	1.000	—	—	—
Dietary habits	0.100*	0.243**	−0.173**	−0.130**	0.171**	−0.139**	−0.071	1.000	—	—
Meal regularity	−0.089*	0.097*	−0.145**	0.063	0.000	0.084*	0.037	0.006	1.000	—
Proactive health literacy	0.201**	−0.169**	0.192**	0.134**	−0.272**	−0.150**	−0.103*	−0.140**	−0.010	1.000

*Data are Spearman’s *ρ*. *p < 0.05, **p < 0.01 (two-sided). *N* = 560 for all variables except education level (*N* = 549).

### 3.5 Multiple linear regression analysis

Separate regression models were conducted with knowledge, belief, behavior, and total health literacy scores as dependent variables, controlling for demographics and lifestyle factors.

Knowledge score: Significant positive predictors included female sex (*β* = 0.195, *p* < 0.001), higher education (*β* = 0.180, *p* = 0.001), and higher monthly income (*β* = 0.165, *p* < 0.001). Significant negative predictors were poorer self-rated health (*β* = −0.156, *p* < 0.001) and unhealthy dietary habits (*β* = −0.120, *p* = 0.005). The results of the multiple linear regression analysis for the health knowledge score are presented in Table 10.

**Table 10:** Multiple linear regression analysis results for the health knowledge score.

Predictor	Unstandardized *β*	SE	Standardized *β*	*t*	*P*-value	95% CI	Tolerance	VIF
Constant	3.066	0.274	—	11.202	<0.0001	2.528 to 3.604	—	—
Sex	0.245	0.062	0.195	3.948	<0.0001	0.123 to 0.366	0.657	1.522
Age group	0.073	0.045	0.088	1.625	0.105	−0.015 to 0.162	0.538	1.858
Education level	0.101	0.029	0.180	3.464	0.001	0.044 to 0.159	0.589	1.699
Monthly household income (participant and spouse only)	0.075	0.019	0.165	3.928	<0.0001	0.038 to 0.113	0.902	1.108
Self-rated health (past year)	−0.109	0.031	−0.156	−3.546	<0.0001	−0.170 to −0.049	0.824	1.214
Smoking status	0.007	0.031	0.011	0.227	0.820	−0.053 to 0.067	0.708	1.411
Alcohol consumption (past year)	−0.004	0.019	−0.009	−0.203	0.839	−0.041 to 0.033	0.814	1.229
Dietary habits	−0.161	0.057	−0.120	−2.828	0.005	−0.274 to −0.049	0.883	1.132
Meal regularity	0.053	0.076	0.029	0.703	0.482	−0.095 to 0.202	0.958	1.044

Outcome variable: Knowledge score (mean). Data are regression coefficients (*β*) with SEs; *p*-values are two-sided. VIF = variance inflation factor. *R*^2^ = 0.133, adjusted *R*^2^ = 0.119.

Belief score: A significant positive predictor was female sex (*β* = 0.249, *p* < 0.001). Significant negative predictors were poorer self-rated health (*β* = −0.175, *p* < 0.001) and unhealthy dietary habits (*β* = −0.117, *p* = 0.007). The results of the multiple linear regression analysis for the health belief and attitude score are presented in Table 11.

**Table 11:** Multiple linear regression analysis results for the health belief and attitude score.

Predictor	Unstandardized *β*	SE	Standardized *β*	*t*	*P*-value	95% CI	Tolerance	VIF
Constant	3.830	0.204	—	18.790	<0.001	3.430 to 4.230	—	—
Sex	0.227	0.046	0.249	4.921	<0.001	0.136 to 0.318	0.657	1.522
Age group	0.029	0.034	0.048	0.866	0.387	−0.037 to 0.095	0.538	1.858
Education level	−0.012	0.022	−0.028	−0.531	0.595	−0.054 to 0.031	0.589	1.699
Monthly household income (participant and spouse only)	0.018	0.014	0.054	1.262	0.207	−0.010 to 0.046	0.902	1.108
Self-rated health (past year)	−0.089	0.023	−0.175	−3.891	<0.001	−0.134 to −0.044	0.824	1.214
Smoking status	0.003	0.023	0.006	0.129	0.897	−0.042 to 0.048	0.708	1.411
Alcohol consumption (past year)	−0.001	0.014	−0.004	−0.094	0.925	−0.029 to 0.026	0.814	1.229
Dietary habits	−0.115	0.043	−0.117	−2.697	0.007	−0.198 to −0.031	0.883	1.132
Meal regularity	0.027	0.056	0.020	0.482	0.630	−0.084 to 0.138	0.958	1.044

Outcome variable: Belief/attitude score (mean). Data are regression coefficients (*β*) with SEs; *p*-values are two-sided. VIF = variance inflation factor. *R*^2^ = 0.082, adjusted *R*^2^ = 0.067.

Behavior score: A significant positive predictor was female sex (*β* = 0.210, *p* < 0.001). Significant negative predictors were poorer self-rated health (*β* = −0.155, *p* = 0.001) and unhealthy dietary habits (*β* = −0.118, *p* = 0.007). The results of the multiple linear regression analysis for the health behavior score are presented in Table 12.

**Table 12:** Multiple linear regression analysis results for the health behavior score.

Predictor	Unstandardized *β*	SE	Standardized *β*	*t*	*P*-value	95% CI	Tolerance	VIF
Constant	3.857	0.243	—	15.858	<0.001	3.379 to 4.335	—	—
Sex	0.230	0.055	0.210	4.180	<0.001	0.122 to 0.338	0.657	1.522
Age group	−0.019	0.040	−0.027	−0.486	0.627	−0.098 to 0.059	0.538	1.858
Education level	0.000	0.026	0.000	0.006	0.995	−0.051 to 0.051	0.589	1.699
Monthly household income (participant and spouse only)	0.033	0.017	0.082	1.914	0.056	−0.001 to 0.066	0.902	1.108
Self-rated health (past year)	−0.094	0.027	−0.155	−3.446	0.001	−0.148 to −0.041	0.824	1.214
Smoking status	−0.004	0.027	−0.007	−0.137	0.891	−0.057 to 0.050	0.708	1.411
Alcohol consumption (past year)	−0.014	0.017	−0.039	−0.856	0.392	−0.047 to 0.019	0.814	1.229
Dietary habits	−0.138	0.051	−0.118	−2.720	0.007	−0.238 to −0.038	0.883	1.132
Meal regularity	0.028	0.067	0.018	0.423	0.672	−0.104 to 0.161	0.958	1.044

Outcome variable: behavior score (mean). Data are regression coefficients (*β*) with SEs; *p*-values are two-sided. VIF = variance inflation factor. *R*^2^ = 0.105, adjusted *R*^2^ = 0.090.

Total health literacy score: Significant positive predictors included female sex (*β* = 0.242, *p* < 0.001) and higher monthly income (*β* = 0.123, *p* = 0.004). Significant negative predictors were poorer self-rated health (*β* = −0.181, *p* < 0.001) and unhealthy dietary habits (*β* = −0.134, *p* = 0.002).

All Variance Inflation Factor (VIF) values were below 2, indicating no multicollinearity. The results of the multiple linear regression analysis for the overall PHL score are presented in Table 13.

**Table 13:** Multiple linear regression analysis results for the overall proactive health literacy score.

Predictor	Unstandardized *β*	SE	Standardized *β*	*t*	*P*-value	95% CI	Tolerance	VIF
Constant	3.886	0.231	—	16.816	<0.001	3.432 to 4.340	—	—
Sex	0.256	0.052	0.242	4.886	<0.001	0.153 to 0.358	0.657	1.522
Age group	0.033	0.038	0.047	0.865	0.387	−0.042 to 0.108	0.538	1.858
Education level	0.036	0.025	0.076	1.448	0.148	−0.013 to 0.084	0.589	1.699
Monthly household income (participant and spouse only)	0.047	0.016	0.123	2.909	0.004	0.015 to 0.079	0.902	1.108
Self-rated health (past year)	−0.107	0.026	−0.181	−4.111	<0.001	−0.158 to −0.056	0.824	1.214
Smoking status	0.003	0.026	0.005	0.100	0.921	−0.048 to 0.053	0.708	1.411
Alcohol consumption (past year)	−0.007	0.016	−0.019	−0.425	0.671	−0.038 to 0.025	0.814	1.229
Dietary habits	−0.151	0.048	−0.134	−3.139	0.002	−0.246 to −0.057	0.883	1.132
Meal regularity	0.040	0.064	0.026	0.631	0.528	−0.085 to 0.166	0.958	1.044

Outcome variable: overall PHL score (mean). Data are regression coefficients (*β*) with SEs; *p*-values are two-sided. VIF = variance inflation factor. *R*^2^ = 0.125, adjusted *R*^2^ = 0.111.

## 4 Discussion

### 4.1 Principal findings

In this community-based cross-sectional survey conducted in Anhui Province, overall PHL was moderate to high, but the domain profile was uneven: Belief scores were the highest, behavior scores were intermediate, and knowledge scores were comparatively lower.

In bivariate analyses, higher PHL was associated with a more favorable sociodemographic and behavioral profile. PHL correlated positively with sex, education, and monthly household income and negatively with age group, smoking, alcohol consumption, unhealthy dietary habits, and poorer self-rated health. Smoking and drinking also tended to co-occur, highlighting the clustering of lifestyle risk factors in this population.

In multivariable models, female sex and higher monthly household income were independently associated with higher overall PHL, whereas poorer self-rated health and unhealthy dietary habits were associated with lower PHL; smoking and alcohol consumption were not retained as independent predictors. In domain-specific models, the knowledge domain was positively associated with female sex, education level, and income, while poorer self-rated health and unhealthy dietary habits were consistent negative predictors. For beliefs and behaviors, female sex remained a positive predictor, and poorer self-rated health and unhealthy dietary habits were consistent negative predictors.

### 4.2 Interpretation in the context of existing evidence

Our findings are broadly consistent with recent evidence syntheses showing that health literacy is positively associated with health-promoting behaviors, although effect sizes are often modest and vary across behavioral domains (4). By focusing on proactive, action-oriented competence—actively seeking information, appraising credibility, and translating knowledge into daily routines—PHL may capture a more behaviorally proximal capability than traditional “functional” literacy measures.

Dietary habits showed the most stable independent association with PHL in the adjusted models, suggesting that diet-related behaviors may be particularly sensitive to differences in proactive health competence. This aligns with empirical research linking higher health literacy and food/nutrition literacy to healthier dietary patterns and better diet quality in adults (5, 9). Conceptually, dietary behavior requires repeated information processing (e.g., understanding dietary guidance and evaluating food choices), planning, and sustained self-regulation; individuals with stronger proactive competence may therefore be better positioned to translate recommendations into everyday eating practices.

Smoking and alcohol consumption were associated with PHL in the correlation analyses, but these associations attenuated after adjustment. This is plausible because tobacco and alcohol use are strongly shaped by gender norms, social contexts, and dependence-related mechanisms, which may reduce the independent contribution of literacy once sociodemographic factors are taken into account (10, 11). Importantly, smoking and drinking often co-occur and reinforce one another, consistent with the co-occurrence observed in this study and with population evidence showing substantial overlap between these behaviors (12). Similar co-use patterns have been described in Chinese rural populations, suggesting that integrated, rather than single-behavior, interventions may be more practical (13). More broadly, behavioral clustering is well documented in contemporary population studies, reinforcing the need for multi-behavior perspectives when interpreting literacy–behavior pathways (14).

PHL was inversely correlated with self-rated health, and the negative coefficient indicates that participants with higher proactive health literacy tended to report better perceived health. This pattern is consistent with evidence linking higher health literacy to more favorable self-rated health outcomes (15, 16). Nevertheless, the cross-sectional design precludes causal inference, and the association may also reflect reverse causation: Individuals experiencing poorer health may engage more actively in information seeking and self-management, which could, in turn, raise their proactive health literacy (17–19). Longitudinal studies that capture baseline morbidity and health information-seeking behaviors are needed to clarify temporal ordering.

Taken together, the gradients by sex and income likely reflect unequal access to learning opportunities, digital and health resources, and supportive environments that enable residents to translate information into sustained action—an interpretation consistent with contemporary research on health literacy inequities (20).

### 4.3 Practical implications

From a public health perspective, the pattern of relatively lower knowledge scores alongside higher belief and behavior scores suggests that community programs should prioritize strengthening actionable knowledge while maintaining motivation and self-efficacy (21). Interventions may be more effective if they emphasize practical competence—such as recognizing misinformation, identifying credible sources, interpreting common health indicators, and navigating local health services—rather than focusing solely on knowledge transmission (22). Given the sociodemographic gradients observed, men and lower-income groups may warrant particular attention to prevent widening inequalities in proactive health management.

The independent role of dietary habits also suggests that diet may be a useful “entry point” for PHL-oriented interventions in community settings (23). Nutrition education may have a greater impact when paired with goal setting, behavioral feedback, and family-based support (24). Meanwhile, because smoking and drinking tended to cluster, integrated approaches that address both behaviors—while linking literacy improvement to concrete cessation or reduction supports—may better reflect real-world behavioral patterns (25).

### 4.4 Strengths and limitations

This study adds community-based evidence on proactive health literacy and its behavioral correlates in a Chinese setting. Several limitations should be acknowledged. First, sample representativeness is limited. Participants were recruited from a single community in Anhui Province and restricted to permanent residents, using a mixed-mode approach (on-site and online questionnaires). Although a simple random sampling strategy was intended, participation was voluntary, and the online component may have preferentially captured individuals with better digital access, higher education, or greater interest in health topics while under-representing those with limited literacy, sensory impairment, or lower engagement. In addition, the study did not use a multi-stage sampling design stratified by urban–rural context or socioeconomic profiles; therefore, the findings should not be extrapolated to other provinces, to settings with different urbanicity, or to mobile/migrant populations without caution.

Importantly, the demographic structure of the sample may further constrain external validity. The study included a relatively high proportion of older adults (approximately 40.4%) and individuals with low education level (approximately 63% reported incomplete primary education or less). Although this distribution may reasonably reflect the characteristics of the sampled community, it differs from the demographic profile of the general Chinese adult population and may limit the replicability and generalizability of the estimates. As both age and education are closely related to health literacy, digital access, and lifestyle behaviors, over-representation of older and less-educated residents could shift the overall level of proactive health literacy and potentially modify the strength of associations between proactive health literacy and lifestyle behaviors. Accordingly, the present findings are most applicable to communities with similar age and education structures, and population-level extrapolation should be made cautiously. Future research should consider multi-site, stratified sampling across settings with different sociodemographic compositions and, where appropriate, post-stratification weighting or age-/education-standardized analyses to improve population-level inference.

Second, the cross-sectional design precludes causal inference and does not allow temporal ordering between proactive health literacy and lifestyle behaviors to be established; reverse causation remains possible (e.g., poorer perceived health motivating more active information seeking and self-management). Third, key variables—including lifestyle behaviors and self-rated health—were self-reported and may be affected by recall error and social desirability bias; using a single survey source also raises the possibility of common-method variance. Fourth, the multiple linear regression models showed modest explanatory power, indicating that a substantial proportion of the variability in proactive health literacy remains unexplained. This suggests that additional individual, interpersonal, and contextual determinants—such as chronic conditions, mental health, motivation, family support, access to primary care, exposure to health communication, and neighborhood food and activity environments—were not fully captured in the current models. Fifth, residual confounding cannot be ruled out because some determinants were not measured in detail, including diagnosed chronic conditions, access to primary care, exposure to health communication, and neighborhood-level environments (e.g., food availability and opportunities for physical activity). Future multi-site longitudinal studies with stratified sampling across urban and rural communities, incorporating more objective behavioral and health measures, are warranted to strengthen generalizability and clarify directionality. Finally, Cronbach’s alpha was very high; although this indicates excellent internal consistency, it may also suggest partial item redundancy. Future validation research could consider item reduction (e.g., factor analytic or IRT/Rasch approaches) to develop a shorter form while preserving construct coverage.

## 5 Conclusion

This study provides empirical evidence on the status of proactive health literacy and lifestyle behaviors among community residents in Anhui, China. Health knowledge is a key area for improvement. Proactive health literacy is significantly associated with demographic factors (sex, education, and income), key lifestyle behaviors (diet, smoking, and drinking), and self-rated health. Targeted, multi-faceted interventions are needed to enhance health literacy, particularly practical knowledge, and to foster the adoption and maintenance of healthy lifestyles, especially among high-risk groups. This is essential for advancing the goals of the “Healthy China” initiative.

## Data Availability

The datasets generated during and/or analysed during the current study are available from the corresponding author on reasonable request.
